# Unconventional Bifunctional Lewis-Brønsted Acid Activation Mode in Bicyclic Guanidine-Catalyzed Conjugate Addition Reactions

**DOI:** 10.3390/molecules200815108

**Published:** 2015-08-18

**Authors:** Bokun Cho, Ming Wah Wong

**Affiliations:** Department of Chemistry, National University of Singapore, 3 Science Drive 3, Singapore 117543, Singapore; E-Mail: BKCho@ntu.edu.sg

**Keywords:** organocatalysis, activation mode, guanidine, bifunctional activation, mechanism, DFT calculations

## Abstract

DFT calculations have demonstrated that the unconventional bifunctional Brønsted-Lewis acid activation mode is generally applicable to a range of nucleophilic conjugate additions catalyzed by bicyclic guanidine catalysts. It competes readily with the conventional bifunctional Brønsted acid mode of activation. The optimal pro-nucleophiles for this unconventional bifunctional activation are acidic substrates with low p*K*_a_, while the best electrophiles are flexible 1,4-diamide and 1,4-diester conjugated systems.

## 1. Introduction

**Scheme 1:**
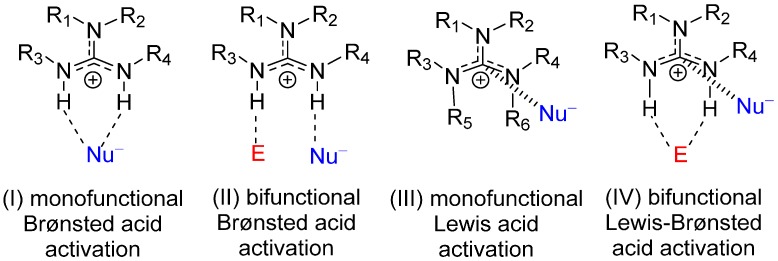
Plausible mono- and bi-functional activation modes in Brønsted base catalysis of guanidine organocatalyst (E = electrophile and ${\text{Nu}}^{-}$ = nucleophile).

Guanidines are versatile organocatalysts with vast applications in asymmetric synthesis [1,2,3,4,5]. The mechanistic aspects of guanidine-catalyzed reactions, in particular the various possible activation modes, have been reviewed recently [1,2]. Due to their strong Lewis basicity (many are in the superbase scale region), bicyclic guanidines are widely used in Brønsted base catalysis. In the Brønsted base mechanism, guanidine abstracts a proton from the substrate and catalyzes the reaction as its conjugate acid, the guanidinium cation. Through hydrogen bonding interactions, the guanidinium ion can either activate the electrophile (mode I, Scheme 1) in a monofunctional mode or both the electrophile and nucleophile simultaneously in a bifunctional manner (mode II, Scheme 1). This bifunctional Brønsted acid activation mode was initially proposed by Corey and Grogan in asymmetric Strecker reactions using a chiral guanidine catalyst [6], and was supported by a theoretical study [7]. X-ray structure of TBD∙HCl∙H_2_O provides further evidence to the bifunctionality of the guanidinium ion [8]. In a very recent kinetic study of three different types of bicyclic guanidine-catalyzed reactions, the bifunctionality of the guanidine catalyst is further established [9]. Our computational studies on bicyclic guanidine-catalyzed Michael [10,11] and phospha-Michael [12] reactions and isomerization reaction of alk-3-ynoates [9] have confirmed this type of bifunctional activation mode. It is worth mentioning that the guanidinium ion also plays an important role in the catalysis of phosphoric ester cleavage [13,14,15,16]. A similar bifunctional general base/general acid mechanism is believed to operate.

**Scheme 2:**
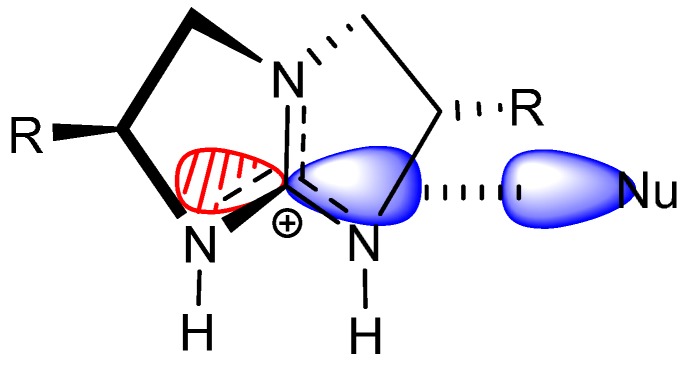
“Lewis acid” interaction between the vacant *p* orbital of the central electrophilic carbon of [5,5] bicyclic guanidinium ion and the lone pair of a nucleophile (Nu).

The guanidinium ion is characterized by a low lying LUMO with a relatively large contribution of a vacant *p* orbital on the central carbon. In addition, the central carbon is strongly positively charged (e.g., NBO charge of +0.74 for [5,5] bicyclic guanidinium) [11,17] similar to that of a typical carbocation. A nucleophile (Lewis base) is expected to interact favorably with the highly electron deficient carbon of the guanidinium ion (Scheme 2). For an anionic nucleophile (e.g., PhS^−^), the strong electrostatic attraction between the central guanidinium carbon and the anion provides an additional source of stabilization. As a result, guanidinium cation could readily serve as a Lewis acid catalyst via the central electrophilic carbon. This lesser known Lewis acid catalytic capability (mode III, Scheme 1) of hexyl alkyl guanidinium salts has been suggested by the authors in catalyzed reactions such as epoxide ring-opening esterification [18], lactide ring-opening polymerization [19] and the decomposition of alkyl formate [20]. Experimental support of such an activation mode is difficult to establish, but there is a wealth of evidence in X-ray crystallography that this type of “Lewis acid” interaction between the guanidinium central carbon and nucleophile exists (see references in Supporting Information).

**Scheme 3:**
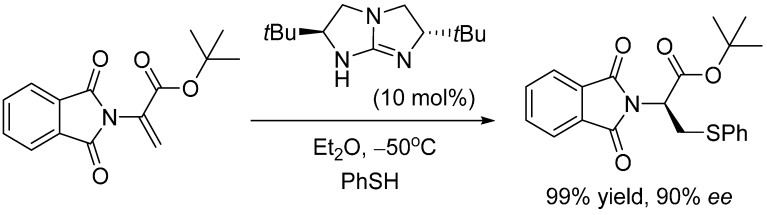
Bicyclic guanidine-catalyzed thio-Michael reaction between thiophenol and phthalimide [21].

One would envisage that this Lewis acid nucleophile activation mode could be combined with the Brønsted acid activation mode of an electrophile to provide an alternate bifunctional activation mode of guanidines as Brønsted base catalysts (mode IV, Scheme 1). Our DFT study [17] of the bicyclic guanidine-catalyzed thio-Michael reaction between thiophenol and phthalimide (Scheme 3) [21] has readily demonstrated this alternate bifunctional Brønsted and Lewis acid activation mode. This unconventional bifunctional mode is also found to play an important catalytic role in our recent computational studies of bicyclic guanidine catalyzed-Michael addition of dimethyl malonate to 2-cyclopenten-1-one [11] and isomerization reaction of alk-3-ynoate [9].

**Scheme 4:**
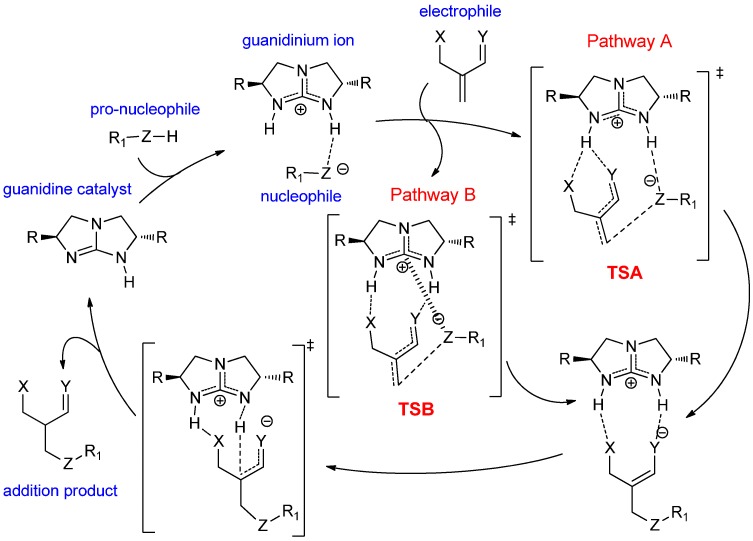
Catalytic cycle of bicyclic guanidine-catalyzed nucleophilic conjugate addition reactions. Pathways A and B are the two possible bifunctional activation modes of guanidinium ion. R_1_-Z-H represents the pro-nucleophile (conjugate base = R_1_-Z^−^) while the X and Y moieties signify the Brønsted basic functional groups of the electrophile.

For the conjugate addition reaction studied [17], this unconventional activation mode, via pathway B (R_1_ = Ph and Z = S, Scheme 4), competes readily with the conventional bifunctional Lewis acid activation mode, via pathway A (Scheme 4). Intriguingly, pathway B determines the enantioselectivity and rate of the reaction. In the catalytic cycle of the thio-Michael addition [17], the guanidine catalyst attracts a proton from thiophenol to form the thiophenolate ion (PhS^−^), which serves as a nucleophile in the subsequent conjugate addition. In the C–S bond forming step, both the electrophile and nucleophile interact with the guanidinium N–H protons simultaneously via hydrogen bond in transition state **TSA** (Scheme 4) in the conventional bifunctional Brønsted acid activation mode (*i.e*., pathway A). On the other hand, the nucleophile interacts with the cationic guanidinium carbon via a “Lewis acid” interaction while the phthalimide electrophile forms dual hydrogen bonds with both the guanidinium N–H protons in transition state **TSB** (Scheme 4) for the unconventional bifunctional Brønsted and Lewis acid activation mode (pathway B). Our computational study [17] showed that pathway B is significantly more favourable when the turnover frequencies of both pathways were compared and is enantioselective towards the *S* product, which agrees well with the experimental observation [21]. In this paper, we explore further the general application of this unconventional bifunctional activation mode to other nucleophilic conjugate additions and elucidate the factors that control the energetic preference towards this intriguing bifunctional activation mode.

## 2. Results and Discussion

### 2.1. Model Systems for Conjugate Nucleophilic Addition

Based on our mechanistic study of the bicyclic guanidine-catalyzed thio-Michael reaction [17], three criteria of the substrates appear to be important for the differential preference of the guanidinium bifunctional Brønsted-Lewis acid activation mode:

(1) The electrophile has two Brønsted basic sites (*i.e*., hydrogen bond acceptors X and Y, see Scheme 4) for dual hydrogen bonding interaction with the two guanidinium N−H protons.
(2) The nucleophile, preferably in an anionic form, should have significant Lewis basicity to interact favorably with the guanidinium central electrophilic carbon, via the “Lewis acid” interaction (Scheme 2).
(3) The electrophile must have sufficient flexibility to enable the nucleophile to access the Lewis acidic site and the unsaturated carbon for conjugation addition simultaneously.

To validate above three criteria, the kinetic preference in the C–Z (where Z = carbon or heteroatom) bond forming transition states of the conventional Brønsted acid activation (pathway A) and the bifunctional Brønsted acid and Lewis activation (pathway B) was explored for nucleophilic addition of several model systems. The kinetic preference ∆∆G^≠^, G(**TSA**)-G(**TSB**), is taken as the difference in Gibbs free energy between the C–Z bond forming transition states of the two possible pathways, namely **TSA** and **TSB** for pathway A and B, respectively. A positive ∆∆G^≠^ implies that **TSB** is more stable than **TSA**, and a preference for pathway B. An unsubstituted [5,5] bicyclic guanidine catalyst (*i.e*., R = H, Scheme 4) was used in this study as our main focus of this paper is on the activation mode, not stereoselectivity. It is important to note that our previous computational study has established that the rate determining step of catalytic Michael reaction is the nucleophilic addition to the carbonyl carbon instead of the protonation step [17]. Based on the super basicity of the bicyclic guanidine together with the known acidities of the pro-nucleophiles considered in the present study, it is unlikely that the activation of reactants is the rate limiting step. Therefore, it is justifiable to apply the Curtin-Hammet principle to the bicyclic guanidine-catalyzed conjugate addition studied here.

### 2.2. Optimal Brønsted Basic Functional Groups

**Figure 1:**
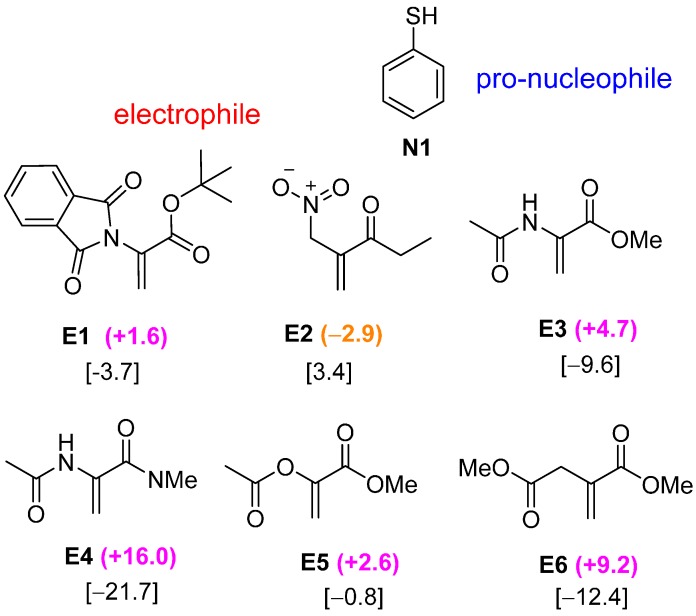
Various electrophiles (**E1**–**E6**) were screened with thiophenol (**N1**) for bicyclic guanidine-catalyzed conjugate addition reactions. ∆∆G^≠^ values (in kJ·mol^−1^) are given in parenthesis and the binding free energies (in kJ·mol^−1^) of the dual hydrogen bonded guanidinium-electrophile complexes are in square bracket.

**Figure 2:**
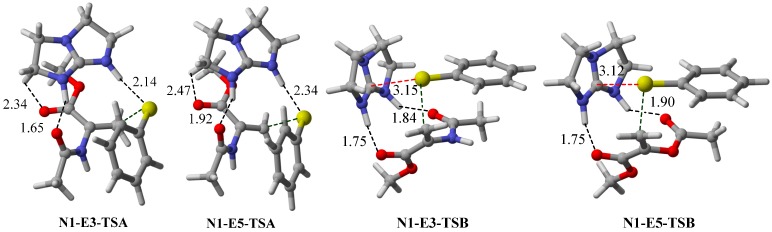
Optimized geometries (M06-2X/6−31G*) of **TSA** and **TSB** for conjugate addition of **E3** and **E5** with **N1**. C∙∙∙S, S∙∙∙H and O∙∙∙H interaction distances are in Å.

Initially, various electrophiles were screened with thiophenol pro-nucleophile (**N1**) to identify which Brønsted basic functional groups are optimal for the bifunctional Lewis and Brønsted acid activation (Figure 1). The thio-Michael reaction of **N1** with electrophile **E1** was elucidated in our previous DFT study [17]. The removal of the *t*-butyl groups on the guanidine catalyst from Scheme 3 reduces the inclination of thio-Michael reaction between **E1** and **N1** to pathway B. Electrophile **E2** was chosen to investigate the effect of absence on *N*-vinyl amide and ester moieties. Using similar nitro and ethyl ketone functional groups, a negative ∆∆G^≠^ value of −2.9 kJ·mol^−1^ was obtained, indicating a preference towards the conventional bifunctional pathway A (*i.e*., Brønsted acid activation). This suggests the importance of the *N*-vinyl amide and ester moiety in favoring the alternate activation mode. When both *N*-vinyl amide and ester groups are used in **E3**, there is a reversal of kinetically preferential towards pathway B, by 4.7 kJ·mol^−1^. Substituting the ester moiety by an amide group (in **E4**) further amplifies the preference of pathway B (∆∆G^≠^ = +16 kJ·mol^−1^). However, replacing the vinyl acetamide in **E3** by a vinyl ester group (in **E5**) does not lead to a significant change in ∆∆G^≠^ (+2.6 kJ·mol^−1^). This may attribute to the fact that the acetamide group possesses a more rigid double bond which restricts the conformational freedom of the electrophile (Figure 2). In other words, **TSB** is somewhat destabilized with respect to **TSA**. The modification of the vinyl acetate to methyl acrylate in **E6** results in a significantly lower energy pathway B (∆∆G^≠^ = +9.2 kJ·mol^−1^). The stronger preference for pathway B is due to the fact that **TSB** for the **N1**-**E6** system possesses stronger hydrogen bonding interaction with the guanidinium ion. This argument is supported by the calculated donor and acceptor interactions in NBO analysis and the atomic charge on the carbonyl oxygen (Supporting Information).

As the unconventional bifunctional Lewis-Brønsted acid activation mode involves dual hydrogen bonding interaction between the two Brønsted basic functional groups (X and Y, Scheme 4) on the electrophile and the two Brønsted acidic N–H protons on the guanidinium ion, the stabilization interaction between the electrophile and guanidinium catalyst is expected to be a key factor for the pathway B activation. This argument is supported by comparing the binding energies of various guanidinium-electrophile hydrogen bonded complexes. The computed binding free energy provides a quantitative measure of the strength of the hydrogen bonding interaction in the guanidinium-electrophile complex. As evidenced in Figure 1, electrophiles **E4** and **E6**, which possess the lowest binding energies, have a strong preference towards pathway B. On the other hand, systems with a low binding energy are associated with less stable **TSB** compared to **TSA**.

It is important to note that noncovalent interactions between the nucleophile and electrophile are less significant compared to the substrate interactions with the guanidinium catalyst. These intermolecular interactions are the weaker C–H∙∙∙X interactions and π–π interactions (see NBO analysis, Supporting Information). As a result, the noncovalent interactions between the electrophile and nucleophile do not have a major impact on the selectivity for the two activation modes.

### 2.3. Optimal Pro-Nucleophiles

**Figure 3:**
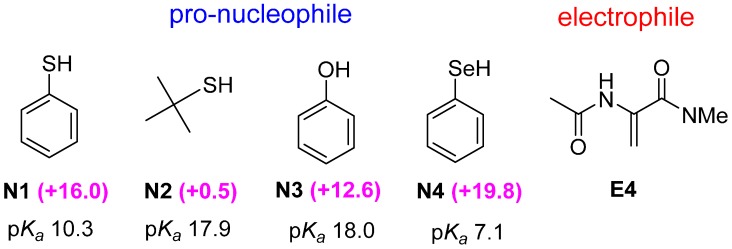
Various pro-nucleophiles (**N1**–**N4**) screened with the optimal electrophile **E4** for bicyclic guanidine-catalyzed conjugate addition reactions. ∆∆G^≠^ values (in kJ·mol^−1^) are given in parenthesis. The p*K_a_* values (in DMSO) were taken from Ref. [22].

Next, we examine optimal pro-nucleophiles for the preference of the bifunctional Brønsted-Lewis acid activation. We employed the best electrophile **E4** to screen with different pro-nucleophiles **N1** to **N4** (Figure 3). As evidence in the large positive ∆∆G^≠^ values (Figure 3), all the phenol analogues are suitable pro-nucleophiles for the preference of pathway B. The trend of ∆∆G^≠^ (**N4** > **N1** > **N3**) indicates that the stability of the conjugate anion play an important role in the energetic preference of pathway B. This is not unexpected as the “Lewis acid” interaction (Scheme 2) involves complexation of the guanidinium cation and conjugate anion (*i.e*., activated nucleophile) of the pro-nucleophile.

Comparison of thiophenol (**N1**) with *t*-butyl thiol (**N2**) further confirms this hypothesis. As a result, the ∆∆G^≠^ value correlates reasonably well with the p*K*_a_ value (in DMSO) [23] of the pro-nucleophile (Figure 3). In general, pro-nucleophile with high acidity (p*K*_a_) has a more stable conjugate base anion, and in turn leads to a more stable transition state (**TSB**) for the bifunctional Brønsted-Lewis activation. For the thiophenol analogues, the aromatic stabilization of the deprotonated aromatic conjugate anion is also a key factor for the energetic preference of pathway B. Since all the nucleophiles considered here are strong nucleophiles in anionic form, the stability of the base anion appears to be a more important factor than the nucleophilicity of the base in influencing the energetic preference towards pathway B, which involves Lewis acid interaction between the base anion and guanidinium ion. The “Lewis acid” interaction is the main source of stabilization in **TSB** type of transition state. The magnitude of this stabilization interaction depends on (1) donor-acceptor interaction between the lone pair of the nucleophile and the vacant *p* orbital of the central guanidinium carbon and (2) electrostatic attraction between the anionic nucleophile and the central carbon of guanidinium, which is essentially a carbocation (NBO charge ~+0.75). For the donor-acceptor interaction, it can be quantified readily by NBO second perturbation analysis (see Table S1, Supporting Information). For instance, the strong preference of **N3-E4-TSB** over **N2-E4-TSB** can be attributed to the relative strength of donor-acceptor interaction, 22.7 *vs.* 2.8 kJ·mol^−1^ (Table S1). The electrostatic attraction between the anionic nucleophile and guanidinium “carbocation” (C^+^) is expected to be large. However, it is less straightforward to quantify this electrostatic stabilization. In the cases of **N2-E4-TSB** and **N3-E4-TSB**, the NBO atomic charges and the anion∙∙∙C^+^ distances (see Figure S1, Supporting Information) clearly demonstrate the significant stronger electrostatic attraction in **N3-E4-TSB**. C–H∙∙∙π stabilization is also evidenced in **N3-E4-TSB** (Figure S1). The different strength of “Lewis acid” interaction together with the additional C–H∙∙∙π stabilization in **N3-E4-TSB** explain the different preference of **TSB** for **N2** and **N3** despite both have nearly the same p*K_a_* values (Figure 3).

Despite being weak conjugate bases, the selected pro-nucleophiles studied here and/or their analogues have been shown to undergo Michael or nucleophilic addition reactions [24,25,26]. Experimental and computational studies of Lewis acid-base adducts by Denmark *et al*., indicated that a Lewis base could enhance the catalytic ability of a Lewis acid [27,28]. When a Lewis acid-base adduct is formed, the attached Lewis base will draw electron density away from the Lewis acid, increasing the partial negative charge on the Lewis base. This dispels the conventional notion that the Lewis base will lose its electron pair and become less nucleophilic. This finding further supports the unconventional bifunctional activation mode via **TSB**.

### 2.4. Bifunctional Lewis-Brønsted Acid Activation in Michael and Hetero-Michael Reactions

**Figure 4:**
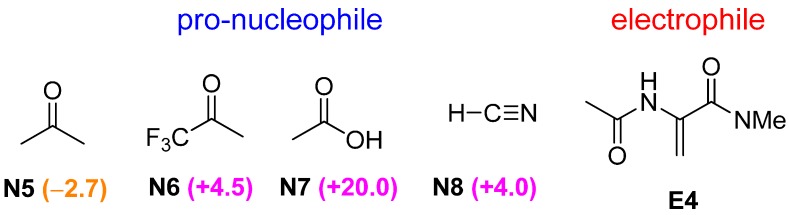
Various acetyl and cyanide nucleophiles screened with the optimal electrophile **E4** for bicyclic guanidine-catalyzed conjugate addition reactions. ∆∆G^≠^ values (in kJ·mol^−1^) are given in parenthesis.

**Figure 5:**
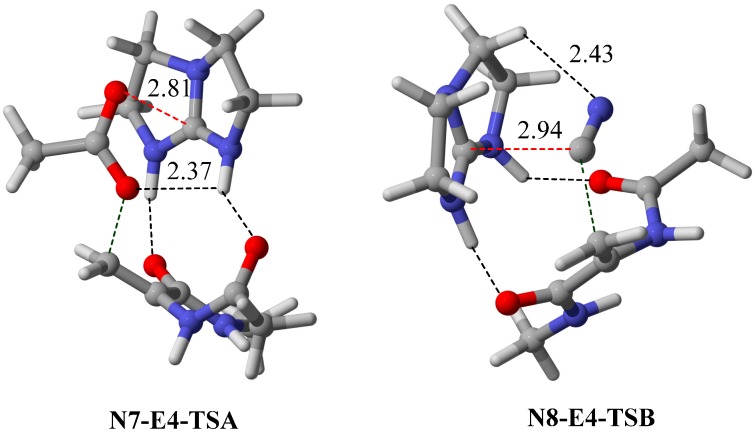
Optimized geometries (M06-2X/6−31G*) of **TSB** transition state for acetate (**N7-E4-TSB**) and cyanide (**N8-E4-TSB**) nucleophiles. Intermolecular interaction distances are given in Å.

To explore further the general applicability of bifunctional Lewis-Brønsted acid mode of activation in Michael and hetero-Michael reactions, pro-nucleophiles **N5**–**N8** were screened with the optimal electrophile **E4** (Figure 4). All the nucleophiles chosen are capable of forming conjugate base anion with resonance stabilization, namely enolates for **N5** and **N6**, acetate for **N7** and cyanide for **N8**. As with several hetero-Michael reactions examined in previous section, the stability of the anion nucleophile is a key factor in governing the selectivity towards pathway B. For instance, substituting acetone (**N5**) with an electron withdrawing triflouromethyl group (in **N6**), increases the acidity of the α-carbon hydrogen [22] and stabilizes the conjugate base enolate ion. As a consequence, there is a reversal of ∆∆G^≠^ on going from **N5** to **N6**. In the same manner, the trend of ∆∆G^≠^ of acetone (**N5**), acetic acid (**N7**) and hydrogen cyanide (**N8**) can readily be explained in terms of the stability of the conjugate base anion. It is worth noting that the transition state **TSB** of acetate nucleophile is strongly favored compared to the cyanide system despite **N7** and **N8** have similar acidity (Figure 5). Interestingly, additional hydrogen bond is found in **TSB** for both cases, C–H∙∙∙N interaction (2.46 Å) between the α-carbon hydrogen with the guanidinium ion in the **N7**–**E4** system and normal O–H∙∙∙O (2.37 Å) hydrogen bond in the **N8**–**E4** case. NBO analysis indicates that the C–H∙∙∙N stabilization energy (1.92 kJ·mol^−1^) in the cyanide system is significantly weaker than the O–H∙∙∙O stabilization (5.36 kJ·mol^−1^) in the acetate system. It is worth noting that Michael addition of HCN with imine corresponds to Strecker reaction and guanidine is one of the most commonly used catalysts for asymmetric synthesis of α-amino acids [29]. Although the convention bifunctional activation mode has been proposed for the guanidine-catalyzed Strecker reactions [6,7], our computational results suggest that the unconventional bifunctional activation mode may play a crucial role in understanding the reactivity of stereoselectivity of guanidine-catalyzed Strecker reactions.

### 2.5. Other Bicyclic Guanidine Catalyst

**Figure 6:**
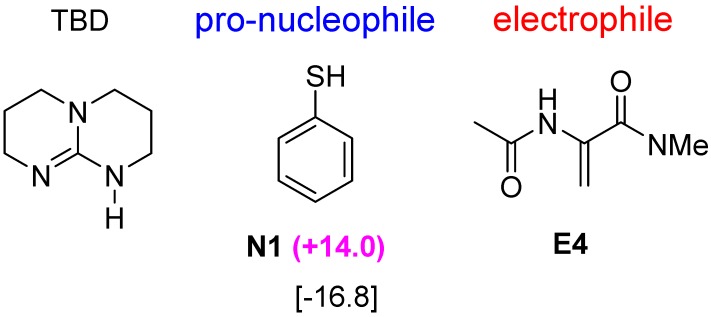
TBD-catalyzed nucleophilic addition between pro-nucleophile **N1** and electrophile **E4**. ∆∆G^≠^ value (in kJ·mol^−1^) is given in parenthesis and binding free energy (in kJ·mol^−1^) is in square bracket.

**Figure 7:**
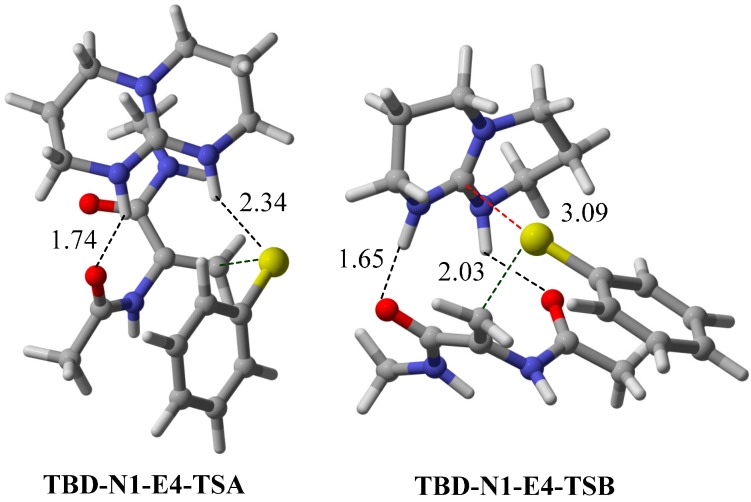
Optimized **TSA** and **TSB** transition states for TBD-catalyzed nucleophilic addition between **N1** and **E4**. Interaction distances are given in Å.

Another popular bicyclic guanidine catalyst, 1,5,7-triazabicyclo[4.4.0]dec-5-ene (TBD), is frequently used in the base catalysis of polymers [30,31], Michael addition [32], Henry [33] and aldol [34] reactions. Thus, we have examined also the TBD-catalyzed conjugate addition of **N1** pro-nucleophile and **E4** electrophile. This 6-membered ring catalyst is predicted to accommodate the bifunctional Lewis-Brønsted acid activation mode favourably (∆∆G^≠^ = +14.0 kJ·mol^−1^, Figure 6). The calculated binding energy of **E4** with TBD is lower than that of the 5-membered ring bicyclic guanidine catalyst. The favourable interaction distances of **TSA** and **TSB** transition states are shown in Figure 7. The strong correlation between higher binding energy and higher selectivity towards the Lewis-Brønsted acid activation mode is again observed in this case. This result suggests that a favourable binding energy provide a good qualitative indication of a favourable Lewis-Brønsted acid activation mode in nucleophilic conjugate addition.

## 3. Computational Methods

### Computational Details

Equilibrium structures and transition states were fully optimized using the M06-2X [35] density functional method together with the standard 6−31G* basis set. The M06-2X functional was chosen as this empirical functional is better suited than normal hybrid DFT methods (e.g., B3LYP) in handling kinetics, thermodynamics, and noncovalent interactions [11,17,35,36,37]. Most importantly, our recent benchmark calculations have shown that dispersion corrections are essential in the DFT functionals to describe the long range “Lewis acid” interaction between a nucleophile and the guanidinium catalyst [17]. Frequency analyses were performed on all M06-2X/6−31G* optimized geometries to confirm the nature of the stationary points as equilibrium structures (with all real frequencies) or transition states (with only one imaginary frequency). The effect of solvation was examined by the SMD [38] implicit solvation model through M06-2X/6-311+G** single-point calculation, based on the gas-phase M06-2X/6−31G* optimized geometry. Both electrostatic and non-electrostatic terms are considered in this solvation calculations. Unless otherwise noted, the relative energies reported in the text correspond to relative free energies at 233 K (ΔG_233_), computed at M06-2X/6-311+G**//M06-2X/6−31G* level in diethyl ether solvent. The relative free energy (Δ*G*_T_) was computed from the equation Δ*G*_T_ = Δ*H*_T_ − *T*Δ*S*, where Δ*S* is the entropy change and Δ*H*_T_ = Δ*H*_0_ + (*H*_T_ − *H*_0_). Natural bond orbital (NBO) analysis was carried out at M06-2X/6−31G* level to study the charge distribution and donor-acceptor interactions, via the second-order perturbation energy analysis [39]. All calculations were performed using the *Gaussian* 09 suite of programs [40].

## 4. Conclusions

In summary, our DFT calculations have demonstrated that the unconventional bifunctional Brønsted-Lewis activation mode of guanidinium catalyst is generally applicable to various nucleophilic conjugate additions. The optimal pro-nucleophiles are acidic substrates with low p*K*_a_ as the resonance stabilization of its conjugate base anion is important. The optimal electrophiles are flexible systems with two Brønsted basic functional groups capable of forming dual hydrogen bond with the guanidinium catalyst. The best candidates for electrophile are 1,4-diamide and 1,4-diester conjugated systems. Since the two possible bifunctional activation modes (modes II and IV, Scheme 1) favor different stereoisomers [17], one can employ the differential stabilization of the two pathways to control the stereoselectivity of nucleophilic addition product.

## Supplementary Materials

Supplementary materials [NBO donor and acceptor interaction analysis, optimized geometries of “**N2-E4-TSB** and **N3-E4-TSB**, references for X-ray structures with guanidinium “Lewis acid” interaction, and Cartesian coordinates of all optimized geometries (in .xyz format)] can be accessed at: http://www.mdpi.com/1420-3049/20/08/15108/s1.
