# Supplementary material for: Unconventional Bifunctional Lewis-Brønsted Acid Activation Mode in Bicyclic Guanidine-Catalyzed Conjugate Addition Reactions
Source: Molecules. 2015 Aug 18;20(8):15108–21. doi: 10.3390/molecules200815108 (PMC6331857; doi:10.3390/molecules200815108)
Supplement: Supplementary file 1 [file molecules-20-15108-s001.pdf]

Supporting Information

Table S1. NBO donor-acceptor interaction analysis for various **TSA** and **TSB** transition states.

TS	Donor	Acceptor	Bond Length/Å	Bond Angle	NBO e2pert/kJ/mol
**N1-E1-TSA**	C=O	N-H	2.06	119	7.2
	C=O	N-H	2.37	111	4.1
	C=O	N-H	2.17	127	6.23
	S	N-H	2.34	135	12.93
**N1-E1-TSB**	C=O	N-H	1.9	150	21.92
	C=O	N-H	1.76	166	37.61
	S	C+	3.2		1.84
**N1-E2-TSA**	C=O	Gua-α-C-H	2.34	130	2.51
	N=O	N-H	1.95	125	11.55
	S	N-H	2.31	153	19.79
**N1-E2-TSB**	C=O	N-H	1.85	151	6.71
	N=O	N-H	2.03	136	12.30
	S	C+	3.15		2.64
**N1-E3-TSA**	C=O	N-H	1.65	157	46.48
	C=O	Gua-α-C-H	2.34	131	2.55
	S	N-H	2.13	170	11.84
**N1-E3-TSB**	C=O	N-H	1.84	144	26.78
	C=O	N-H	1.75	158	37.07
	S	C+	3.15		5.31
**N1-E4-TSA**	C=O	N-H	1.62	163	52.59
	C=O	Gua-α-C-H	2.28	127	3.22
	S	N-H	2.23	170	9.79
**N1-E4-TSB**	C=O	N-H	1.90	139	19.08
	C=O	N-H	1.63	163	49.04
	S	C+	3.16		5.90
**N1-E5-TSA**	C=O	N-H	1.92	135	16.86
	C=O	N-H	2.30	116	6.78
	C=O	Gua-α-C-H	2.47	120	1.42
	S	N-H	2.34	135	13.39
	C=O	C=C	3.14		1.92
**N1-E5-TSB**	C=O	N-H	1.90	142	23.39
	C=O	N-H	1.75	155	37.66
	S	C+	3.12		6.44
	C=O	C=C	3.30		1.26
**N1-E6-TSA**	C=O	N-H	1.94	134	14.56
	C=O	N-H	2.29	118	5.65
	C=O	Gua-α-C-H	2.45	120	1.55
	S	N-H	2.43	131	10.21
	C=O	C=C	3.30		1.21
**N1-E6-TSB**	C=O	N-H	1.93	138	19.79
	C=O	N-H	1.69	156	44.89
	S	C+	3.11		6.95

Table S1. *Cont.*

TS	Donor	Acceptor	Bond Length/Å	Bond Angle	NBO e2pert/kJ/mol
**N2-E4-TSA**	C=O	N-H	1.70	159	46.94
	C=O	Gua-α-C-H	2.26	134	3.60
	S	N-H	2.10	171	12.84
	N	C-H	2.76	160	2.80
**N2-E4-TSB**	C=O	N-H	1.90	140	20.88
	C=O	N-H	1.67	165	46.02
	S	C+	3.10		5.65
**N3-E4-TSA**	C=O	N-H	1.65	166	49.66
	C=O	Gua-α-C-H	2.33	127	2.43
	O	N-H	1.58	174	8.87
	C=O	C=C	3.02		2.26
**N3-E4-TSB**	C=O	N-H	1.89	145	20.79
	C=O	N-H	1.70	170	38.16
	O	C+	2.69		22.72
**N4-E4-TSA**	C=O	N-H	1.95	125	20.79
	C=O	N-H	1.72	157	36.94
	Se	N-H	2.71	119	4.73
**N4-E4-TSB**	C=O	N-H	1.92	139	19.00
	C=O	N-H	1.64	163	47.15
	Se	C+	3.10		8.28
**N5-E4-TSA**	C=O	N-H	1.88	149	30.38
	C=O	Gua-α-C-H	2.57	121	1.26
	O	C-H	2.29	136	8.41
**N5-E4-TSB**	C=O	N-H	1.94	139	18.87
	C=O	N-H	1.76	165	35.61
	O	C+	2.63		6.99
**N6-E4-TSA**	C=O	N-H	1.99	129	12.43
	C=O	Gua-α-C-H	2.17	130	6.23
	O	N-H	1.87	135	12.55
	O	N-H	2.13		12.22
	F	N-H	2.50	106	1.00
**N6-E4-TSB**	C=O	N-H	1.95	140	18.95
	C=O	N-H	1.70	165	42.80
	O	C+	2.61		11.42
	F	N-H	2.47	110	1.26
**N7-E4-TSA**	C=O	N-H	1.85	151	24.64
	C=O	Gua-α-C-H	2.28	133	2.01
	O=C=O	N-H	1.70	161	25.98
	O=C=O	N-H	2.43	109	1.38
	O=C=O	N-H	2.38	139	7.86

Table S1. *Cont.*

TS	Donor	Acceptor	Bond Length/Å	Bond Angle	NBO e2pert/kJ/mol
**N7-E4-TSB**	C=O	N-H	1.93	145	22.13
	C=O	N-H	1.69	170	47.99
	O=C=O	N-H	2.37	107	5.36
	O=C=O	C+	2.81		1.38
**N8-E4-TSA**	C=O	N-H	2.24	112	5.56
	C=O	Gua-α-C-H	2.34	119	2.43
	C≡N	N-H	2.08	132	7.07
	C≡N	N-H	2.17	126	8.79
**N8-E4-TSB**	C=O	N-H	1.69	167	41.09
	C=O	N-H	1.86	143	25.06
	C≡N	Gua-α-C-H	2.46	115	1.92
	C≡N	Gua-α-C-H	2.64	105	0.84
	N≡C	C+	2.94		2.59
	C≡N	C-H	2.91	118	1.63
**TBD_N1-E4-TSA**	C=O	N-H	1.73	145	35.48
	C=O	Gua-α-C-H	2.34	132	2.85
	S	N-H	2.34	148	8.33
**TBD_N1-E4-TSB**	C=O	N-H	2.03	132	11.55
	C=O	N-H	1.65	173	42.93
	S	C+	3.09		8.66

NBO e2pert is the donor and acceptor interaction energy of the NBO Lewis bonding molecular orbitals to the antibonding molecular orbitals; **Donor**: S = thiolate sulphur atom, O = anionic O atom, C=O = carbonyl O atom, O=C=O = carboxylate O atom, N≡C = cyanide C atom, C≡N = cyanide N atom, C=C is π donation from ethene or aromatic moiety, F= fluorine atom; **Acceptor**: N-H = guanidinium N-H hydrogen, Gua-α-C-H = C-H interaction of hydrogen on α carbon w.r.t to N of guanidine, C+ = central carbon atom in the guanidinium functional group, Ph-H = hydrogen on phenyl ring, C-H = hydrogen from methylene group; The interactions in black are between the catalyst with both electrophile and nucleophile while that in red are between the electrophile and nucleophile.

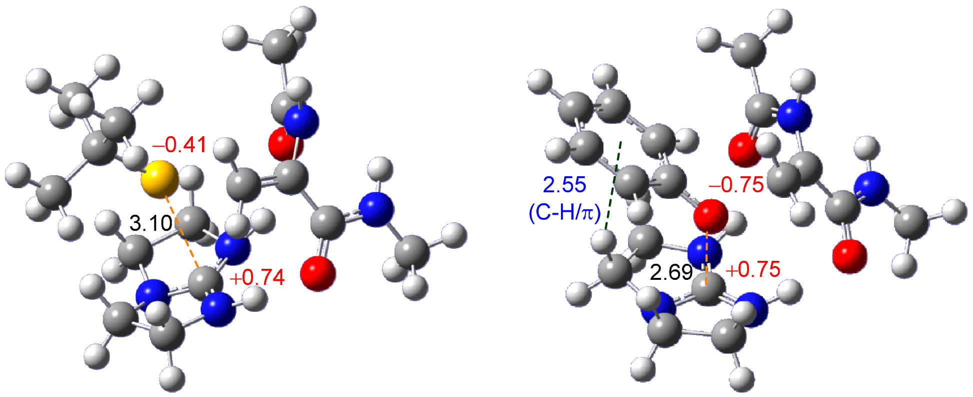

N2-E4-TSB **N3-E4-TSB**

Figure S1. Optimized (M06-2X/6−31G*) geometries of **N2-E4-TSB** and **N3-E4-TSB**. NBO atomic charges are in red. Interaction distances are in Å.

References for X-ray Structures with $uanidinium "Lewis acid" Interaction

1. Wild, U.; Roquette, P.; Kaifer, E.; Mautz, J.; Hubner, O.; Wadepohl, H.; Himmel, H.J. The First Metal Complexes of the Proton Sponge 1,8-Bis(*N,N,N',N'*-tetramethylguanidino)naphtalene: Synthesis and Properties. *Eur. J. Inorg. Chem.* **2008**, *28*, 1248–1257.
2. Soria, D.B.; Grundy, J.; Coles, M.P.; Hitchcock, P.B. Stabilisation of high oxidation-state niobium using "electron-rich" bicyclic-guanidinates. *J. Organomet. Chem.* **2005**, *690*, 2278–2284.
3. Huczynski, A.; Pospieszny, T.; Ratajczak-Sitarz, M.; Katrusiak, A.; Brzezinski, B. Structural and spectroscopic studies of the 1:1 complex of lasalocid acid with 1,5,7-triazabicyclo[4.4.0]dec-5-ene. *J. Mol. Struct.* **2008**, *875*, 501–508.
4. Sawada, M.; Tanaka, T.; Furukawa, Y.; Toda, T.; Tadai, Y.; Hanafusa, T. X-ray Structure Determination and NMR Characterization of Some Fused Heterocycles with 1,3,5-Triazine-2,4 (1*H*,3*H*)-dione Ring. Reaction of 2-Amino-4(3*H*)-pyrimidinone with Chloroformyl Isocyanoate. *Bull. Chem. Soc. Jpn.* **1988**, *61*, 2217–2219.
5. He, H.Y.; Faulkner, D.J. A New Imidazole Alkaloid from the Marine Sponge Leucetta microrhaphis. *J. Org. Chem.* **1992**, *57*, 2176–2178.
6. Xiao, L.X.; Shi, D.Q. 3-[(2-Chlorothiazol-5-yl)methyl]-5-(isobutylamino)-6-phenyl-3*H*-1,2,3-triazolo-[4,5-d]pyrimidin-7(6*H*)-one. *Acta Cryst.* **2007**, *E63*, o2843.
7. Neuba, A.; Herres-Pawlis, S.; Flörke, U, Henkel, G. *Z. N*-[Bis(dimethylamino)methyl-idene]-2-[(triphenylmethyl)sulfanyl]ethanaminium hexafluorophosphate. *Anorg. Allg. Chem.* **2008**, *634*, 771–777.
8. Elawn, N.M.; Awad, E.M.; Hassaneen, H.M.; Linden, A.; Heimgartner, H. Synthesis of 1,2,4-Triazolo[4,3-a]pyrimidine Derivatives by Cyclocondensation of a 2-Thioxopyrimidin-4(3*H*)-one with Hydrazonoyl. *Helv. Chim. Acta* **2003**, *86*, 739–749.
9. Polienko, J.F.; Schanding, T.; Gatilov, Y.V.; Grigor'ev, I.A.; Voinov, M.A. Studies toward the Synthesis of 4-(2-*R*-ethyl)amino-2,2,5,5-tetramethyl-3-imidazoline 1-Oxyls. Nucleophilic Substitution of Bromide in the *N*-Alkyl Chain of the 1,2,4-Oxadiazol-2-one Precursor. *J. Org. Chem.* **2008**, *73*, 502–510.
10. Karolak-Wojciechowska, J.; Kieć-Kononowicz, K. 5,5-Diphenyl-1*H*,2*H*,5*H*-imidazo-[1,2-a] limidazole-3,6-dione. *Acta Cryst.* **1994**, *C50*, 300–302.
11. Kieć-Kononowicz, K.; Müller, C.E.; Pekala, E.; Karolak-Wojciechowska, J.; Handzlik, J.; Łażewska, D. Imidazo[2,1-*b*]thiazoles, Imidazo[2,1-*b*]imidazoles and Pyrrolo[1,2-*c*]imidazoles. Synthesis, Structure and Evaluation of Benzodiazepine Receptor Binding. *J. Heterocycl. Chem.* **2002**, *39*, 243–253.
12. Takanyanagi, H.; Ogura, H.; Tsuzuno, N.; Kubota, I.; Iitaka, Y. Molecular Structure of 3,4,6-Tri-*O*-acetyl-1,2-*O*-[(1*S*)-1-(2-p-bromophenyl)-9*H*-imidazo[1,2-a]benzimidazol-3-yl]ethylidene]-α-D-glucopyranose Acetone Solvate. *Bull. Chem. Soc. Jpn.* **1983**, *56*, 3537–3538.
